# *CD300C* reduces lung adenocarcinoma susceptibility through regulation of CD62L⁻ monocytes: a Mendelian randomization study

**DOI:** 10.1007/s12672-026-04481-8

**Published:** 2026-01-24

**Authors:** Huiling Chen, Zhichun Xue, Liwen Huang, Ying Zeng, Meiyan Tang, Kunhuang Han, Jia Chen, Xinyu Deng, Guiju Fang

**Affiliations:** 1 College of Marine Sciences, Ningde Normal University, Ningde, China; 2 Department of Respiratory and Critical Care Medicine, Ningde Municipal Hospital of Ningde Normal University, Ningde, Fujian China; 3 Clinical laboratory, Ningde Clinical Medical College of Fujian Medical University, Ningde, China; 4 Department of painology, Ningde Clinical Medical College of Fujian Medical University, Ningde, China; 5 Engineering Research Center of Mindong Aquatic Product Deep-Processing, Ningde, China; 6 Ningde Yiye Marine Industry Development Co., Ltd, Ningde, China

**Keywords:** CD300C, CD62L⁻ monocytes, Lung adenocarcinoma, Mendelian randomization, Multi-omics integration

## Abstract

**Background:**

This exploratory, hypothesis-generating study aimed to evaluate the potential genetically informed association between *CD300C* gene expression and lung adenocarcinoma (LUAD) risk, and to investigate the possible mediating role of CD62L⁻ monocytes using a multi-omics Mendelian randomization (MR) framework.

**Methods:**

We integrated LUAD GWAS summary statistics, peripheral blood eQTL and pQTL data, and transcriptomic profiles. Candidate genes were prioritized by overlapping evidence from eQTL-MR, pQTL-MR, and expression analyses. A three-step Mendelian randomization model estimated the total, mediated, and direct effects of *CD300C* expression on LUAD risk via CD62L⁻ monocytes.

**Results:**

*CD300C* was the only gene consistently associated with LUAD across all omics stages. Higher *CD300C* expression was associated with reduced LUAD risk (*β* = − 0.030, OR = 0.97, 95% CI: 0.942–0.999), while the proportion of CD62L⁻ monocytes was also inversely associated with LUAD (*β* = − 0.086, OR = 0.91, 95% CI: 0.855–0.982). *CD300C* expression was positively associated with CD62L⁻ monocytes (*β* = 0.008, OR = 1.082, 95% CI: 1.029–1.139). The estimated mediated effect was β = -0.007, accounting for 22.92% of the total association.

**Conclusions:**

Our findings provide preliminary, genetically informed evidence that higher *CD300C* expression may be nominally associated with reduced LUAD risk, potentially in part through CD62L⁻ monocytes. Given the limited statistical power and the lack of significance after multiple-testing correction, these findings should be interpreted as exploratory and hypothesis-generating. They nominate the *CD300C*–CD62L⁻ monocyte axis as a hypothesis for future investigation.

**Supplementary Information:**

The online version contains supplementary material available at 10.1007/s12672-026-04481-8.

## Introduction

Lung adenocarcinoma (LUAD), the most common subtype of non-small cell lung cancer (NSCLC), accounts for approximately 40% of all lung cancer cases [1]. Despite advances in surgery, chemoradiotherapy, and targeted treatments, the five-year survival rate remains below 20%, largely because of high heterogeneity, frequent relapse, and therapeutic resistance. These challenges underscore LUAD as a major global health burden [2, 3]. However, the molecular mechanisms driving LUAD progression, particularly those that enable immune evasion, remain incompletely understood. Recent research has increasingly focused on immunoregulatory factors within the tumor microenvironment, especially immune-related gene expression, and their potential role in cancer development [4]. A deeper understanding of the immunogenetic landscape of LUAD may help uncover novel therapeutic targets and guide the development of more effective immunomodulatory strategies.

Immune regulation has emerged as a central focus in LUAD research [5]. The tumor immune microenvironment (TIME), comprising various immune cell types such as tumor-associated macrophages (TAMs), dendritic cells (DCs), T lymphocytes, and myeloid-derived suppressor cells (MDSCs), plays a critical role in tumor initiation, progression, and response to immunotherapy [6]. Among these, M2-polarized macrophages promote angiogenesis and secrete immunosuppressive cytokines, thereby facilitating immune evasion and tumor advancement [7]. In parallel, metabolic reprogramming, particularly lipid metabolism, profoundly shapes immune cell phenotypes, favoring M2 polarization and impairing T cell-mediated antitumor activity, ultimately diminishing the effectiveness of immunotherapy [8]. Additionally, novel forms of programmed cell death such as ferroptosis have been implicated in LUAD immune regulation and are associated with patient prognosis. Despite increasing insights into the function of immune cell subsets in TIME, their upstream molecular regulators remain poorly defined. In particular, the mechanisms governing the abundance and activation states of distinct immune subpopulations are not well understood [9, 10]. The absence of genetic evidence for causal immune modulators hinders identification of effective targets for immune-based therapies.

Mendelian randomization (MR) is a genetic epidemiological approach that leverages germline variants as instrumental variables to strengthen causal inference while reducing confounding and reverse causation by mimicking randomized controlled trials [11, 12]. In recent years, MR has been widely applied in immunogenetics and cancer research to explore the causal pathways between molecular traits and disease outcomes. Several studies have reported causal links between genetically predicted exposure to radiation and cancer risk. For example, Kim et al. identified positive associations between serum magnesium levels and breast cancer, vitamin B12, and colorectal cancer [13]. Li et al. found that elevated cathepsin H levels were linked to increased LUAD risk, whereas Zhu et al. reported associations between C-reactive protein (CRP) levels and multiple cancer types, including lung cancer, highlighting the potential role of immune-related biomarkers in tumorigenesis [14, 15]. Despite these advances, most MR studies have focused on direct exposure–outcome relationships. Whether immunoregulatory genes influence tumor susceptibility through specific immune cell subsets remains largely unexplored. In particular, the role of *CD300C*, an innate immunoregulatory receptor, in modulating LUAD risk via immune cell composition has not been evaluated from a causal genetic perspective.

Here, we applied a multi-omics MR framework integrating GWAS, eQTL, pQTL, and transcriptomic data to investigate the potential role of *CD300C*, an innate immunoregulatory receptor, in LUAD susceptibility. We further aimed to evaluate whether its effect might be mediated through the proportion of CD62L⁻ monocytes using a three-step MR model. These analyses were designed to provide genetically informed insights into the immunogenetic regulation of LUAD and to assess whether the *CD300C*–monocytes axis may represent a candidate pathway for future mechanistic and translational investigation.

## Methods

### Design

This study was conducted using a multi-omics MR framework to explore genetically informed pathways and putative causal relationships among gene expression, immune cell traits, and LUAD risk. These represent statistical causal inferences under MR assumptions, rather than direct experimental causality. To minimize confounding and ensure the validity of our findings, all MR analyses in this study were required to meet three fundamental assumptions [16]: (1) the instrumental variables (IVs) must be robustly associated with the exposure; (2) the IVs must be independent of any confounding factors; and (3) the IVs must affect the outcome only through the exposure of interest.

**Fig. 1:**
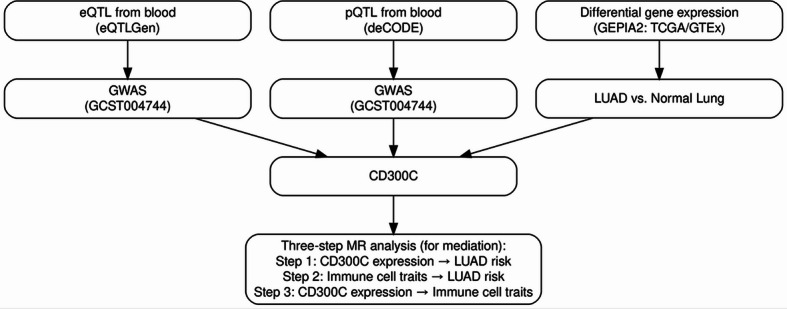
Overview of the multi-omics Mendelian randomization (MR) framework

In particular, we applied a three-step Mendelian randomization (Three-Step MR) mediation analysis, which enables the evaluation of the indirect (mediated) and direct pathways linking genetic regulation to disease risk. In this framework, gene expression was set as the exposure, immune cell traits as potential mediators, and LUAD as the outcome (Fig. 1).

### Data sources and ethical considerations

eQTL data were obtained from the eQTLGen Consortium (https://www.eqtlgen.org/cis-eqtls.html), based on whole blood samples. Plasma pQTL summary statistics were downloaded from the deCODE database (https://www.decode.com/summarydata/). Genetic association data for immune cell traits were sourced from the IEU OpenGWAS project (ebi-a-GCST90001391 to ebi-a-GCST90002121, https://gwas.mrcieu.ac.uk/). GWAS summary statistics for lung adenocarcinoma (LUAD) were obtained from the GWAS Catalog (GCST004744, https://www.ebi.ac.uk/gwas/studies/GCST004744). All datasets used were publicly available and approved by their respective institutional review boards [17–19]. Because both eQTL and pQTL data are primarily derived from blood-based samples, they may not fully capture tissue- or cell type–specific regulatory effects. To address this limitation, we additionally validated immune cell–specific expression patterns using LUAD single-cell RNA-seq datasets (Sect. 2.9). Further sensitivity analyses incorporating lung tissue cis-eQTLs from GTEx v10 are described in the Supplementary Methods (Section S1).

### Instrument selection and quality control

SNPs were selected as instrumental variables (IVs) according to genome-wide significance (*p* < 5 × 10⁻⁸). To ensure independence, we performed LD clumping with a 10,000 kb window and an *r²* threshold of < 0.001, as recommended by recent studies. Only SNPs with *F*-statistics > 10 were retained to mitigate weak instrument bias. Palindromic and ambiguous SNPs were excluded. All instrumental variables used in the MR analysis underwent rigorous screening to ensure their validity, including exclusion of SNPs associated with known confounders or the outcome itself, as identified through the LDtrait Tool and GWAS Catalog. Only after this multi-step filtering were candidate genes with robust MR associations considered meaningful for further analysis [20].

### MR analysis and sensitivity testing

In the MR analysis, the Wald ratio method was applied when the exposure was instrumented using a single SNP. For exposures associated with multiple SNPs, causal effects were primarily estimated using the inverse-variance weighted (IVW) method. To detect and account for potential horizontal pleiotropy, we implemented several robust MR approaches, including MR-Egger regression, the weighted median, simple mode, and weighted mode, to assess the consistency of causal estimates under different assumptions. These complementary approaches provide effect estimates under different model assumptions and help to assess the robustness of the findings. Leave-one-out (LOO) analysis was additionally performed under the IVW framework, in which each SNP was sequentially excluded from the instrument set to evaluate whether the overall causal estimate was disproportionately driven by a single variant. The MR-Egger intercept test was used to assess the directional pleiotropy. Cochran’s Q test and the Heterogeneity in Dependent Instruments (HEIDI) test were employed to evaluate potential heterogeneity among the selected IVs. A non-significant Cochran’s Q statistic (*P* > 0.05) indicated low heterogeneity, thereby supporting the reliability of MR estimates [20, 21]. Post hoc statistical power was evaluated using the mRnd online tool (https://shiny.cnsgenomics.com/mRnd/), parameterized by the LUAD GWAS (*N* = 66,756; case proportion = 0.1689), the variance explained by *CD300C* instruments (R² ≈ 0.03–0.05), and the observed effect size (OR = 0.97 per SD). The estimated power was approximately 10–13% at α = 0.05 and ≈ 0% under Bonferroni correction (Table S8). Colocalization analysis was conducted using the coloc R package to assess whether *CD300C* cis-eQTLs and LUAD GWAS signals shared a common causal variant, with posterior probabilities (PP.H4) estimated for evidence of colocalization.

### Differential expression analysis

Transcriptomic data from LUAD tumors and normal lung tissues were analyzed using the GEPIA2 platform, integrating the TCGA and GTEx datasets. Differential expression analysis was performed using thresholds of |log₂ fold change (FC)| > 1 and FDR-adjusted *q* value < 0.05. One-way ANOVA was used to confirm the statistical differences between the tissue types [22].

### Differential gene prioritization

To refine the candidate genes supported by converging evidence, we intersected those identified as significantly differentially expressed with genes showing significant associations in the MR analysis. Genes for which higher expression was genetically predicted to be associated with increased LUAD risk and that were upregulated in tumor tissue were considered putative oncogenic candidates. Conversely, genes with predicted protective associations and concomitant downregulation in tumors were classified as potentially tumor suppressive.

### Three-step MR analysis

To investigate whether the association between *CD300C* expression and LUAD risk may be mediated by immune cell traits, we implemented a three-step MR framework consisting of the following causal paths:*CD300C* expression → LUAD risk: The total effect of *CD300C* expression (instrumented by its cis-eQTL) on LUAD risk was estimated. Immune cell traits → LUAD risk: GWAS summary statistics from the IEU OpenGWAS database were used to estimate the associations between specific immune cell subsets and LUAD risk. *CD300C* expression → Immune cell traits: We assessed whether *CD300C* expression was genetically associated with the proportion of relevant immune cell subsets. we also performed exploratory mediation analyses for other immune cell subsets that showed suggestive associations with both *CD300C* expression and LUAD risk, including plasmacytoid dendritic cells (plasmacytoid DC AC) and CD28⁺CD45RA⁺CD8⁺ T cells. Independent SNPs from immune cell GWAS datasets were selected as instruments. The total effect (*β*_total), the effect of *CD300C* on immune traits (*β*₁), and the effect of immune traits on LUAD risk (*β*₂) were estimated. The indirect (mediated) effect was calculated as *β*_indirect = *β*₁ × *β*₂, and the mediation proportion was estimated as proportion mediated (%) = (*β*_indirect / *β*_total) × 100% [23].

### Single-cell RNA-seq analysis

To further validate the phenotype of CD62L⁻ monocytes in the LUAD tumor microenvironment, we analyzed publicly available non-small cell lung cancer (NSCLC) single-cell RNA sequencing (RNA-seq) datasets enriched for LUAD from the Tumor Immune Single-cell Hub 2 (TISCH2) portal (primary dataset: NSCLC_GSE131907; validation dataset: NSCLC_GSE117570). Uniform Manifold Approximation and Projection (UMAP)–based cell clustering and curated cell-type annotations were obtained from TISCH2. Expression levels of *SELL* (CD62L), pro-inflammatory markers (*CX3CR1*, *FCGR3A*), and immunoregulatory markers (*MRC1*, *CD16*3) were visualized using feature and violin plots.

## Results

### Instrument selection and data filtering

After quality control and harmonization, a total of 15,695 genes with eQTL data, 1,615 proteins with pQTL data, and 612 immune cell traits were retained for further analysis. The number of valid SNPs and detailed instrumental variable information for candidate genes, proteins, and immune cell traits included in the MR analyses are provided in Supplementary Tables S1, S2, and S4.

### Genetic association between gene expression and LUAD risk

**Fig. 2:**
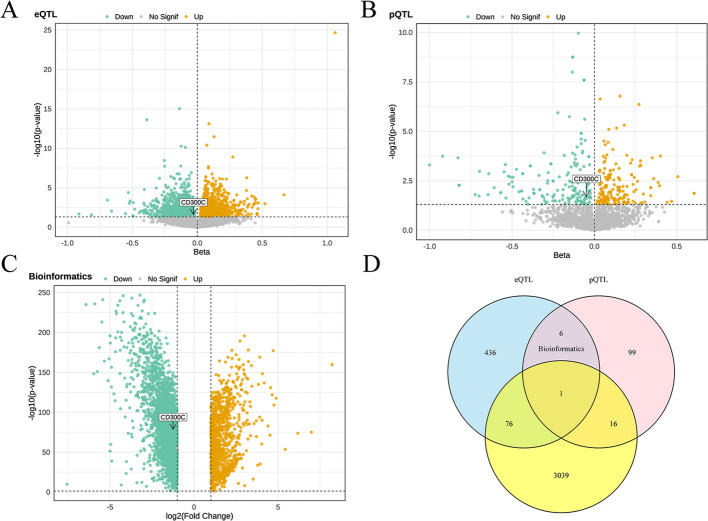
Multi-omics identification of candidate genes associated with LUAD. (A)eQTL-MR, (B) pQTL-MR, and (C) transcriptomic differential expression volcano plots highlight genes associated with LUAD risk. (D) Venn diagram showing overlapping genes among the three analyses.

To identify genes with potential causal effects on LUAD risk, we performed MR analysis by integrating blood-derived eQTL data with LUAD GWAS summary statistics from a European cohort. The exposure (eQTL/pQTL) and outcome (LUAD GWAS) datasets were derived from independent studies with no sample overlap, thereby minimizing bias. Using the IVW method, 1,034 genes were found to be statistically significant (*P* < 0.05). Of these, higher expression of 519 genes was associated with decreased LUAD risk, whereas 515 genes were associated with increased risk. Sensitivity analyses indicated that the majority of these associations showed no substantial heterogeneity (*P* > 0.05; Table S1; Fig. 2A). A sensitivity analysis using GTEx lung tissue eQTLs did not identify significant associations (Supplementary Results, Section S1).

### Proteome-level genetic associations with LUAD risk

To evaluate potential associations at the proteomic level, we performed independent two-sample MR analysis using plasma pQTL data and LUAD GWAS summary statistics. A total of 281 protein loci showed significant associations, including 122 proteins whose increased levels were associated with reduced LUAD risk and 159 with increased risk. Sensitivity analyses indicated low heterogeneity for these associations (*P* > 0.05; Table S2; Fig. 2B).

### Tissue-specific expression of candidate genes in LUAD

To evaluate transcriptional differences in LUAD, we performed differential expression analysis using the Gene Expression Profiling Interactive Analysis 2 (GEPIA2) platform. A total of 4,236 genes were significantly altered, including 3,132 downregulated and 1,104 upregulated in tumor tissues (Table S3; Fig. 2C).

### Cross-omics integration of candidate genes

To prioritize genes supported by convergent multi-omics evidence, we intersected the results from eQTL-MR, pQTL-MR, and transcriptomic differential expression analyses. After applying stringent multiple-testing correction, *CD300C* did not pass the conservative Bonferroni thresholds or the global FDR threshold (*q* ≈ 1). Because many molecular traits are highly correlated, the effective number of independent tests is likely smaller than assumed by conventional Bonferroni and FDR procedures. Consequently, while the *CD300C* association did not surpass these stringent thresholds, we interpret it as a nominal and exploratory signal that is consistently supported across all three omics layers, making *CD300C* the only gene identified in eQTL-MR, pQTL-MR, and transcriptomic analyses (Fig. 2D). Consistent with the modest instrument strength and small effect size, post hoc power was limited (~ 10–13% at α = 0.05 and ≈ 0% after Bonferroni correction), indicating that the *CD300C*–LUAD association should be interpreted as exploratory (Table S8). Colocalization analysis between *CD300C* cis-eQTLs and LUAD GWAS signals yielded a posterior probability for a shared causal variant (PP.H4) of 0.029, indicating limited evidence for colocalization and suggesting that the observed association is unlikely to be solely driven by linkage disequilibrium. In the eQTL-MR analysis, genetically predicted higher *CD300C* expression was nominally associated with reduced LUAD risk (*β* = − 0.03, OR = 0.97, 95% CI: 0.94–0.999; *P* = 0.043; Table S1). Effect estimates were consistent across alternative MR methods (MR-Egger, weighted median, weighted mode, simple mode; Supplementary Table S6), with no evidence of heterogeneity (Cochran’s Q = 50.945, *P* = 0.593) or directional pleiotropy (MR-Egger intercept = 0.0012, *P* = 0.861). Leave-one-out analyses further confirmed that no single SNP disproportionately influenced the association (Fig. S1). Similarly, in the pQTL-MR analysis, genetically elevated *CD300C* protein levels were associated with lower LUAD risk (*β* = − 0.049, OR = 0.95, 95% CI: 0.91–0.99; *P* = 0.025; Table S2), with consistent results across sensitivity analyses (Cochran’s Q = 97.744, *P* = 0.403; MR-Egger intercept = − 0.009, *P* = 0.056). Transcriptomic differential expression analysis further showed that *CD300C* was significantly downregulated in LUAD tumor tissues compared with normal lung tissues (log₂ fold change = − 1.244; *P* = 6.7 × 10⁻⁷⁷; Table S3).

### Mediation analysis of *CD300C* expression and LUAD risk via CD62L⁻ monocytes

**Fig. 3:**
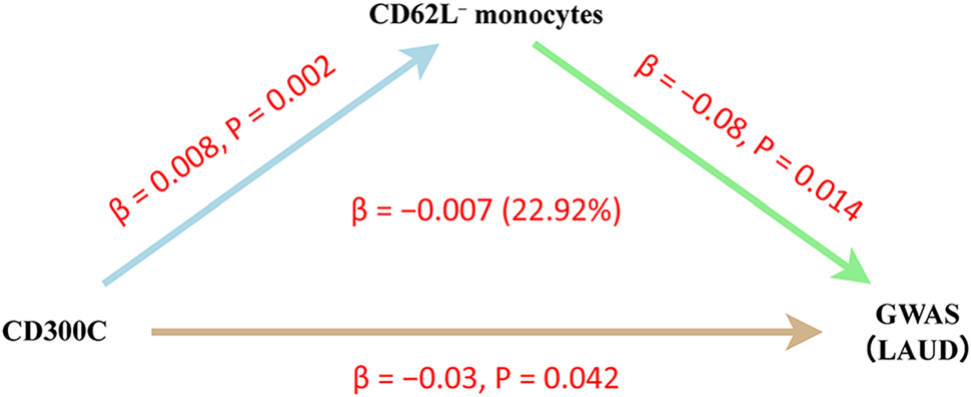
Three-step Mendelian randomization analysis of the *CD300C*–CD62L⁻ monocytes–LUAD pathway

We applied a three-step MR framework to evaluate whether the genetically predicted association between *CD300C* expression and LUAD risk is partially mediated by CD62L⁻ monocytes. In step 1, genetically predicted higher *CD300C* expression was nominally associated with reduced LUAD risk (*β* = − 0.030, OR = 0.97, 95% CI: 0.942–0.999; Table S1). In step 2, a higher proportion of CD62L⁻ monocytes was associated with reduced LUAD risk (*β* = − 0.086, OR = 0.91, 95% CI: 0.855–0.982; Table S4). In step 3, *CD300C* expression was positively associated with the proportion of CD62L⁻ monocytes (*β* = 0.008, OR = 1.082, 95% CI: 1.029–1.139; Table S5). The estimated indirect effect was β = − 0.007, accounting for ~ 22.9% of the total effect (Fig. 3 and Table S7).

### Exploratory mediation analyses of additional immune cell subsets

To examine whether other immune cell subsets might also mediate the association between *CD300C* expression and LUAD risk, we conducted additional three-step MR analyses for pDCs and CD28⁺CD45RA⁺CD8⁺ T cells. Although these subsets showed nominal associations with *CD300C* expression and/or LUAD risk (Table S7), their estimated indirect effects were minimal and not statistically significant (*β* = 0.092 and − 0.192). These findings suggest that CD62L⁻ monocytes remain the most plausible immune mediator of the *CD300C*–LUAD association among the tested subsets.

### Single-cell RNA-seq validation

**Fig. 4:**
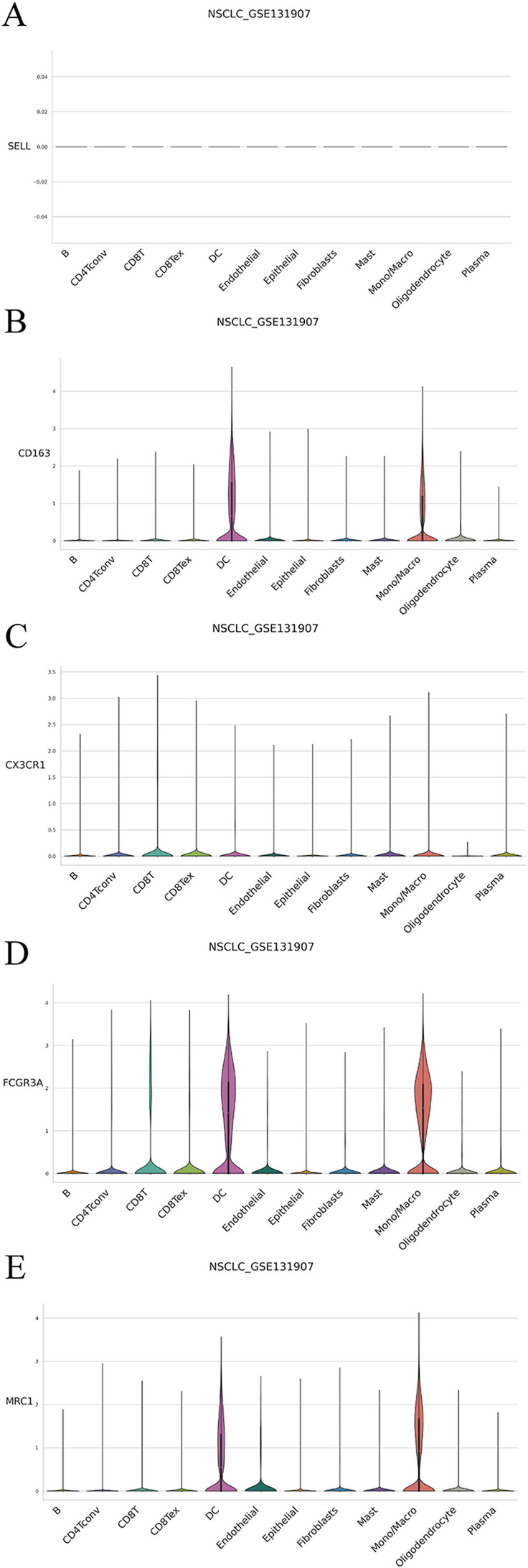
Expression of *SELL*, *CX3CR1*, *FCGR3A*, *MRC1*, and *CD163* across major cell types in LUAD single-cell RNA-seq data

In the NSCLC_GSE131907 dataset, the monocyte/macrophage cluster consistently exhibited low SELL (CD62L) expression, confirming a CD62L⁻ phenotype. Notably, these CD62L⁻ cells displayed a CX3CR1^hi and FCGR3A^hi transcriptional profile, together with low or absent expression of the immunoregulatory markers MRC1 and CD163 (Fig. 4 and Fig. S2). This expression pattern corresponds to a pro-inflammatory, patrolling-like monocyte state rather than an M2-like immunosuppressive phenotype. These single-cell characteristics support the MR findings by suggesting that genetically influenced CD62L⁻ monocytes may possess an intrinsically pro-inflammatory and potentially anti-tumor profile in peripheral blood, before any tumor microenvironment–driven repolarization occurs.

## Discussion

In this exploratory, hypothesis-generating study, we applied a multi-omics Mendelian randomization framework to investigate the potential association between *CD300C* expression and genetic susceptibility to LUAD. By integrating eQTL, pQTL, and transcriptomic data, *CD300C* consistently emerged as a candidate gene associated with reduced LUAD risk. A three-step MR analysis further indicated that this association may be partly mediated by CD62L⁻ monocytes. These findings provide preliminary genetic evidence suggesting a potential immunoregulatory pathway linking gene regulation, immune cell composition, and LUAD susceptibility, and highlight the potential relevance of the *CD300C*–monocyte axis for future mechanistic studies. While exploratory in nature, our results nominate *CD300C* as a promising candidate that warrants replication in independent datasets and functional validation. These results, together with the limited post hoc power (< 13% at α = 0.05 and ≈ 0% after Bonferroni correction), reinforce that our findings are exploratory and hypothesis-generating rather than confirmatory.

*CD300C* is a member of the CD300 receptor family and is predominantly expressed in monocytes and other myeloid cells [24, 25]. It functions as an activating immune receptor by associating with the adaptor FcRγ, which contains an immunoreceptor tyrosine-based activation motif (ITAM) [26]. This interaction triggers calcium influx and NF-κB activation, thereby promoting the expression of costimulatory molecules and the release of pro-inflammatory cytokines such as TNF-α, IL-1β, and IL-6 [27–29]. *CD300C* signaling can synergize with Toll-like receptor pathways, amplifying inflammatory responses without inducing IL-10, suggesting its role as a pro-inflammatory amplifier within the innate immune system. At the same time, *CD300C* has been proposed to act as a co-inhibitory ligand of the B7 family, highlighting its dual functionality in balancing immune homeostasis [30, 31].

Building on these signaling properties, our findings support a working model in which *CD300C* influences LUAD susceptibility by modulating CD62L⁻ monocytes. *CD300C* engagement may promote shedding of L-selectin (CD62L) and expansion of non-classical CD14+ /CD16 + monocytes, a patrolling subset with enhanced migratory capacity [32, 33]. In the lung, these cells have been implicated in antitumor immunity through CCL21-mediated CD8⁺ T cell recruitment and antigen presentation [35]. Conversely, within the tumor microenvironment, CD62L⁻ monocytes may differentiate into immunosuppressive macrophages that express PD-L1 or secrete IL-10 and TGF-β, thereby facilitating immune evasion [34–37]. This context-dependent plasticity suggests that genetically predicted upregulation of *CD300C* may shift the balance toward an antitumor phenotype in LUAD, though direct functional validation remains essential.

Taken together, these observations allow a coherent interpretation of the seemingly divergent roles of CD62L⁻ monocytes. Our MR analyses capture the genetically determined, intrinsic state of circulating CD62L⁻ monocytes, which—supported by our single-cell findings (CX3CR1^hi, FCGR3A^hi, MRC1^lo, CD163^lo)—exhibit a patrolling, pro-inflammatory phenotype with potential anti-tumor activity. In contrast, the immunosuppressive and pro-tumoral behavior reported in previous studies likely reflects secondary reprogramming induced by the tumor microenvironment rather than a primary genetically encoded property [34–37]. Thus, our results support an upstream model in which genetic predisposition promotes the generation of CD62L⁻ monocytes with greater anti-tumor potential in peripheral blood, whereas their later polarization within the tumor microenvironment represents a downstream, context-dependent process. This framework reconciles the protective MR association with prior reports of pro-tumoral macrophage differentiation in LUAD.

We considered CD62L⁻ monocytes as a mediator because *CD300C* is predominantly expressed in myeloid cells, and among 612 immune cell t raits, this subset was the only one significantly associated with both *CD300C* expression and LUAD risk. To assess specificity, exploratory mediation analyses were also conducted for plasmacytoid dendritic cells and CD28⁺CD45RA⁺CD8^bright T cells, but neither showed significant mediated effects (β_indirect = 0.0058 and − 0.0028, both *P* > 0.08). This supports the specificity of CD62L⁻ monocytes as a plausible mediator linking *CD300C* expression to LUAD susceptibility.

Additional biological support was provided by single-cell RNA-seq data from LUAD-enriched NSCLC cohorts (TISCH2 portal), in which monocyte/macrophage clusters exhibited low *SELL* expression, consistent with the CD62L⁻ phenotype, along with high expression of pro-inflammatory markers (*CX3CR1*,* FCGR3A*) and minimal expression of immunoregulatory markers (*MRC1*,* CD163*) [32, 33]. This convergence of genetic and transcriptomic evidence provides both statistical and biological justification for prioritizing CD62L⁻ monocytes in the *CD300C*–LUAD pathway.

Our three-step MR framework relies on core assumptions that the genetic instruments are strongly associated with the exposure, are independent of potential confounders, and influence the outcome only through the exposure. Multiple sensitivity analyses—including MR-Egger regression, weighted median, and leave-one-out testing—supported the robustness of the causal estimates with no evidence of heterogeneity or directional pleiotropy.Moreover, the colocalization analysis between *CD300C* cis-eQTLs and LUAD GWAS signals yielded limited evidence for a shared causal variant (PP.H4 = 0.029). This suggests that the observed association is unlikely to be solely driven by linkage disequilibrium, although residual LD or unmeasured confounding cannot be fully excluded. These considerations collectively reinforce that our findings should be interpreted as exploratory and hypothesis-generating.

## Strengths and limitations

This study has several strengths. First, we applied a multi-omics MR framework integrating large-scale eQTL, pQTL, and transcriptomic data, thereby enhancing statistical power and reducing the likelihood of false positives. Second, by employing complementary MR approaches (IVW, MR-Egger, weighted median) together with sensitivity analyses, we increased confidence in the robustness of our findings. Third, to our knowledge, this is the first study to investigate the potential mediating role of CD62L⁻ monocytes in the *CD300C*–LUAD association using a three-step MR design, thereby offering novel genetically informed insights into immune regulation. Furthermore, we triangulated our findings with single-cell RNA-seq datasets, thereby strengthening their biological plausibility.

Several limitations should also be acknowledged. First, the association between *CD300C* and LUAD risk did not withstand stringent Bonferroni or FDR correction. Given the strong correlation structure among omics traits, such adjustments may be overly conservative, and our findings should therefore be considered exploratory. Second, the genetic association data were predominantly derived from individuals of European ancestry, which may limit generalizability to other populations. Third, the analyses relied on summary-level statistics, precluding individual-level stratification or interaction analyses. Fourth, although multiple sensitivity analyses reduced concerns about pleiotropy, residual horizontal pleiotropy cannot be entirely excluded. Fifth, immune cell traits and eQTL instruments were primarily derived from peripheral blood, which may not fully capture myeloid regulatory programs within the LUAD tumor microenvironment. This tissue-context discrepancy is more likely to attenuate true associations than to generate spurious ones. To address this concern, we conducted sensitivity analyses using GTEx v10 lung cis-eQTLs and triangulated our results with LUAD single-cell RNA-seq data. Although the GTEx analysis was underpowered due to limited sample size and bulk tissue averaging, the overall consistency across datasets supports both robustness and biological plausibility. Sixth, colocalization analysis between *CD300C* cis-eQTLs and LUAD GWAS signals did not provide strong evidence for a shared causal variant (PP.H4 = 0.029), most likely reflecting limited statistical power and differences in linkage disequilibrium, and should therefore be interpreted with caution. The post hoc power for detecting the observed effect size (OR = 0.97 per SD) was < 13% at α = 0.05 and ≈ 0% after Bonferroni correction, underscoring the exploratory nature of our findings. Finally, the causal inferences from MR and mediation analyses should be regarded as genetically informed evidence rather than direct experimental validation; functional studies will be required to confirm the underlying mechanisms.

## Conclusions

In conclusion, this study applied a multi-omics Mendelian randomization (MR) framework to investigate the potential association between *CD300C* expression and genetic susceptibility to LUAD. By integrating eQTL, pQTL, and transcriptomic data, *CD300C* was consistently identified as a candidate gene, and a three-step MR analysis suggested that this association may be partially mediated by CD62L⁻ monocytes. These findings provide preliminary, genetically informed evidence suggesting a potential immunoregulatory pathway linking gene regulation, immune cell composition, and LUAD susceptibility. However, given the limited post hoc power and nominal significance, these results should be interpreted as exploratory and hypothesis-generating rather than confirmatory. Importantly, even modest genetic effects can be biologically meaningful; for example, IL6R variants with similarly small effect sizes have guided the development of IL-6–targeted therapies [38–40]. Nevertheless, our findings should be interpreted with caution given the limitations of MR, including multiple-testing burden, tissue-context differences, and the lack of colocalization support. In this context, these preliminary findings should be interpreted as exploratory and hypothesis-generating. They nominate the *CD300C*–CD62L⁻ monocyte axis as a hypothesis for future investigation, requiring replication in larger datasets.

## Supplementary Information

Below is the link to the electronic supplementary material.

Supplementary Material 1. Figure S1. Leave-one-out MR sensitivity analyses for the associations among *CD300C*, CD62L⁻ Monocytes, and LUAD. (A) *CD300C* expression (eQTL-based instruments) → LUAD. (B) *CD300C* protein levels (pQTL-based instruments) → LUAD. (C) *CD300C* expression → CD62L⁻ Monocytes. (D) CD62L⁻ Monocytes → LUAD.Each point represents the IVW estimate after exclusion of one SNP, with the dashed line indicating the overall IVW estimate using all SNPs. Across all analyses, exclusion of individual SNPs did not materially alter the effect estimates, suggesting that no single SNP disproportionately drove the associations.

Supplementary Material 2. Figure S2. UMAP visualization of *SELL*,* CX3CR1*,* FCGR3A*,* MRC1*, and *CD163* expression in LUAD single-cell RNA-seq data.

Supplementary Material 3. Table S1.Two-sample MR analysis of blood eQTLs associated with LUAD (*P* < 0.05). Table S2. Two-sample MR analysis of blood pQTLs associated with LUAD (*P* < 0.05). Table S3. Differentially expressed genes between LUAD tumor and normal tissues (GEPIA2). Table S4. MR estimates of immune cell and LUAD risk. Table S5. MR analysis of *CD300C* expression and immune cell traits. Table S6. Summary of MR sensitivity analyses for *CD300C* across eQTL, pQTL, and immune traits. Table S7. Three-step Mendelian randomization mediation analyses of immune cell subsets in the *CD300C*–LUAD association. Table S8. Post hoc power analysis of the *CD300C*–LUAD Mendelian randomization association.

## Data Availability

All datasets used in this study were obtained from publicly available resources, including the eQTLGen Consortium, deCODE Genetics, and published LUAD GWAS datasets. Processed data and analysis scripts supporting the findings of this study are available at: https://github.com/YINJUNXUE/CD300C_LUAD_MRAdditional information is available from the corresponding author upon reasonable request.

## Abbreviations

LUAD: Lung adenocarcinoma
MR: Mendelian randomization
GWAS: Genome-wide association study
eQTL: Expression quantitative trait locus
pQTL: Protein quantitative trait locus
SNP: Single nucleotide polymorphism
IV: Instrumental variable
OR: Odds ratio
CD62L⁻: L-selectin negative (CD62L-negative)

## References

[1] Chen Y, Tang L, Huang W, Zhang Y, Abisola FH, Li L. Identification and validation of a novel cuproptosis-related signature as a prognostic model for lung adenocarcinoma. Front Endocrinol (Lausanne). 2022;13:963220. 10.3389/fendo.2022.963220. 36353226 10.3389/fendo.2022.963220 PMC9637654

[2] Ren Q, Zhang P, Lin H, Feng Y, Chi H, Zhang X, Xia Z, Cai H, Yu Y. A novel signature predicts prognosis and immunotherapy in lung adenocarcinoma based on cancer-associated fibroblasts. Front Immunol. 2023;14:1201573. 10.3389/fimmu.2023.1201573. 37325647 10.3389/fimmu.2023.1201573 PMC10264584

[3] Sung H, Ferlay J, Siegel RL, Laversanne M, Soerjomataram I, Jemal A, Bray F. Global cancer statistics 2020: GLOBOCAN estimates of incidence and mortality worldwide for 36 cancers in 185 countries. CA Cancer J Clin. 2021;71(3):209–49. 10.3322/caac.21660. 33538338 10.3322/caac.21660

[4] Liang J, Bi G, Huang Y, Zhao G, Sui Q, Zhang H, Bian Y, Yin J, Wang Q, Chen Z, Zhan C. MAFF confers vulnerability to cisplatin-based and ionizing radiation treatments by modulating ferroptosis and cell cycle progression in lung adenocarcinoma. Drug Resist Updat. 2024;73:101057. 10.1016/j.drup.2024.101057. 38266355 10.1016/j.drup.2024.101057

[5] Wang C, Yu Q, Song T, Wang Z, Song L, Yang Y, Shao J, Li J, Ni Y, Chao N, Zhang L, Li W. The heterogeneous immune landscape between lung adenocarcinoma and squamous carcinoma revealed by single-cell RNA sequencing. Signal Transduct Target Ther. 2022;7(1):289. 10.1038/s41392-022-01130-8. 36008393 10.1038/s41392-022-01130-8 PMC9411197

[6] Mellman I, Chen DS, Powles T, Turley SJ. The cancer-immunity cycle: indication, genotype, and immunotype. Immunity. 2023;56(10):2188–205. 10.1016/j.immuni.2023.09.011. 37820582 10.1016/j.immuni.2023.09.011

[7] Wang Y, Zhang J, Shi H, Wang M, Yu D, Fu M, et al. M2 tumor-associated macrophage–derived Exosomal MALAT1 promotes Glycolysis and gastric cancer progression. Adv Sci (Weinh). 2024;11(24):e2309298. 10.1002/advs.202309298. 38639382 10.1002/advs.202309298 PMC11199979

[8] Park J, Hsueh PC, Li Z, Ho PC. Microenvironment-driven metabolic adaptations guiding CD8⁺ T cell anti-tumor immunity. Immunity. 2023;56(1):32–42. 10.1016/j.immuni.2022.12.008. 36630916 10.1016/j.immuni.2022.12.008

[9] Huang Z, Chen X, Wang Y, Yuan J, Li J, Hang W, et al. SLC7A11 inhibits ferroptosis and downregulates PD-L1 levels in lung adenocarcinoma. Front Immunol. 2024;15:1372215. 10.3389/fimmu.2024.1372215. 38655266 10.3389/fimmu.2024.1372215 PMC11035808

[10] Li Z, Lu W, Yin F, Zeng P, Li H, Huang A. Overexpression of TNFSF11 reduces GPX4 levels and increases sensitivity to ferroptosis inducers in lung adenocarcinoma. J Transl Med. 2024;22(1):340. 10.1186/s12967-024-05112-y. 38594779 10.1186/s12967-024-05112-y PMC11005202

[11] Yeung SLA, Luo S, Iwagami M, Goto A. Introduction to Mendelian randomization. Ann Clin Epidemiol. 2025;7(1):27–37. 10.37737/ace.25004. 39926273 10.37737/ace.25004 PMC11799858

[12] Sekula P, Del Greco MF, Pattaro C, Köttgen A. Mendelian randomization as an approach to assess causality using observational data. J Am Soc Nephrol. 2016;27(11):3253–65. 10.1681/ASN.2016010098. 27486138 10.1681/ASN.2016010098 PMC5084898

[13] Kim JY, Song M, Kim MS, Natarajan P, Do R, Myung W, Won HH. An atlas of associations between 14 micronutrients and 22 cancer outcomes: Mendelian randomization analyses. BMC Med. 2023;21(1):316. 10.1186/s12916-023-03018-y. 37605270 10.1186/s12916-023-03018-y PMC10441703

[14] Li J, Tang M, Gao X, Tian S, Liu W. Mendelian randomization analyses explore the relationship between cathepsins and lung cancer. Commun Biol. 2023;6(1):1019. 10.1038/s42003-023-05408-7. 37805623 10.1038/s42003-023-05408-7 PMC10560205

[15] Zhu M, Ma Z, Zhang X, Hang D, Yin R, Feng J, Xu L, Shen H. C-reactive protein and cancer risk: a pan-cancer study of prospective cohort and Mendelian randomization analysis. BMC Med. 2022;20(1):301. 10.1186/s12916-022-02506-x. 36117174 10.1186/s12916-022-02506-x PMC9484145

[16] Lawlor DA, Harbord RM, Sterne JA, Timpson N, Davey Smith G. Mendelian randomization: using genes as instruments for making causal inferences in epidemiology. Stat Med. 2008;27(8):1133–63. 10.1002/sim.3034. 17886233 10.1002/sim.3034

[17] Võsa T, Claringbould A, Westra H-J, et al. Unraveling the polygenic architecture of complex traits using blood eQTL meta-analysis. Nat Genet. 2018;50(5):1–13. 10.1038/s41588-018-0081-7.29273803

[18] Ferkingstad E, Sulem P, Atlason BA, et al. Large-scale integration of the plasma proteome with genetics and disease. Nat Genet. 2021;53(12):1712–21. 10.1038/s41588-021-00978-w. 34857953 10.1038/s41588-021-00978-w

[19] Orrù V, Steri M, Sidore C, et al. Complex genetic signatures in immune cells underlie autoimmunity and inform therapy. Nat Genet. 2020;52(10):1036–45. 10.1038/s41588-020-0684-4. 32929287 10.1038/s41588-020-0684-4 PMC8517961

[20] Burgess S, Thompson SG. Interpreting findings from Mendelian randomization using the MR-Egger method. Eur J Epidemiol. 2017;32(5):377–89. 10.1007/s10654-017-0255-x. 28527048 10.1007/s10654-017-0255-x PMC5506233

[21] Burgess S, Bowden J, Fall T, Ingelsson E, Thompson SG. Sensitivity analyses for robust causal inference from Mendelian randomization analyses using multiple genetic variants. Epidemiology. 2017;28(1):30–42. 10.1097/EDE.0000000000000559. 27749700 10.1097/EDE.0000000000000559 PMC5133381

[22] Tang Z, Kang B, Li C, Chen T, Zhang Z. GEPIA2: an enhanced web server for large-scale expression profiling and interactive analysis. Nucleic Acids Res. 2019;47(W1):W556–60. 10.1093/nar/gkz430. 31114875 10.1093/nar/gkz430 PMC6602440

[23] Sanderson E. Multivariable Mendelian randomization and mediation. Cold Spring Harb Perspect Med. 2021;11(2):a038984. 10.1101/cshperspect.a038984. 32341063 10.1101/cshperspect.a038984 PMC7849347

[24] Zenarruzabeitia O, Vitallé J, Eguizabal C, Simhadri VR, Borrego F. The biology and disease relevance of CD300a, an inhibitory receptor for phosphatidylserine and phosphatidylethanolamine. J Immunol. 2015;194(11):5053–60. 10.4049/jimmunol.1500304. 25980030 10.4049/jimmunol.1500304

[25] Voss OH, Tian L, Murakami Y, Coligan JE, Krzewski K. Emerging role of CD300 receptors in regulating myeloid cell efferocytosis. Mol Cell Oncol. 2015;2(4):e964625. 10.4161/23723548.2014.964625. 27308512 10.4161/23723548.2014.964625 PMC4905414

[26] Takahashi M, Izawa K, Kashiwakura JI, et al. Human *CD300C* delivers an Fc receptor-γ-dependent activating signal in mast cells and monocytes and differs from CD300A in ligand recognition. J Biol Chem. 2013;288(11):7662–75. 10.1074/jbc.M112.434746. 23372157 10.1074/jbc.M112.434746 PMC3597807

[27] Simhadri VR, Mariano JL, Gil-Krzewska A, Zhou Q, Borrego F. *CD300c* is an activating receptor expressed on human monocytes. J Innate Immun. 2013;5(4):389–400. 10.1159/000350523. 23571507 10.1159/000350523 PMC6741618

[28] Liu N, Sun W, Gao W, et al. CD300e: emerging role and mechanism as an immune-activating receptor. Int Immunopharmacol. 2024;133:112055. 10.1016/j.intimp.2024.112055. 38677094 10.1016/j.intimp.2024.112055

[29] Avlas S, Kassis H, Itan M, et al. CD300b regulates intestinal inflammation and promotes repair in colitis. Front Immunol. 2023;14:1050245. 10.3389/fimmu.2023.1050245. 37033950 10.3389/fimmu.2023.1050245 PMC10073762

[30] Cui C, Su M, Lin Y, Lai L. A *CD300c*-Fc fusion protein inhibits T cell immunity. Front Immunol. 2018;9:2657. 10.3389/fimmu.2018.02657. 30498497 10.3389/fimmu.2018.02657 PMC6249344

[31] Buckle I, Guillerey C. Inhibitory receptors and immune checkpoints regulating natural killer cell responses to cancer. Cancers (Basel). 2021;13(17):4263. 10.3390/cancers13174263. 34503073 10.3390/cancers13174263 PMC8428224

[32] Ziegler-Heitbrock L. The CD14⁺ CD16⁺ blood monocytes: their role in infection and inflammation. J Leukoc Biol. 2007;81(3):584–92. 10.1189/jlb.0806510. 17135573 10.1189/jlb.0806510

[33] Cros J, Cagnard N, Woollard K, et al. Human CD14^dim^ monocytes patrol and sense nucleic acids and viruses via TLR7 and TLR8 receptors. Immunity. 2010;33(3):375–86. 10.1016/j.immuni.2010.08.012. 20832340 10.1016/j.immuni.2010.08.012 PMC3063338

[34] Italiani P, Boraschi D. From monocytes to M1/M2 macrophages: phenotypical vs. functional differentiation. Front Immunol. 2014;5:514. 10.3389/fimmu.2014.00514. 25368618 10.3389/fimmu.2014.00514 PMC4201108

[35] Hanna RN, Cekic C, Sag D, et al. Patrolling monocytes control tumor metastasis to the lung. Science. 2015;350(6263):985–90. 10.1126/science.aac9407. 26494174 10.1126/science.aac9407 PMC4869713

[36] Auffray C, Sieweke MH, Geissmann F. Blood monocytes: development, heterogeneity, and relationship with dendritic cells. Annu Rev Immunol. 2009;27:669–92. 10.1146/annurev.immunol.021908.132557. 19132917 10.1146/annurev.immunol.021908.132557

[37] Randolph GJ, Jakubzick C, Qu C. Antigen presentation by monocytes and monocyte-derived cells. Curr Opin Immunol. 2008;20(1):52–60. 10.1016/j.coi.2007.10.010. 18160272 10.1016/j.coi.2007.10.010 PMC2408874

[38] Visscher PM, Wray NR, Zhang Q, et al. 10 years of GWAS discovery: biology, function, and translation. Am J Hum Genet. 2017;101(1):5–22. 10.1016/j.ajhg.2017.06.005. 28686856 10.1016/j.ajhg.2017.06.005 PMC5501872

[39] Ferreira RC, Freitag DF, Cutler AJ, et al. Functional IL6R 358Ala allele impairs classical IL-6 receptor signaling and influences risk of diverse inflammatory diseases. PLoS Genet. 2013;9(4):e1003444. 10.1371/journal.pgen.1003444. 23593036 10.1371/journal.pgen.1003444 PMC3617094

[40] IL6R Mendelian Randomisation Analysis (IL6R MR) Consortium, Swerdlow DI, Holmes MV, Kuchenbaecker KB, et al. The interleukin-6 receptor as a target for prevention of coronary heart disease: a Mendelian randomisation analysis. Lancet. 2012;379(9822):1214–24. 10.1016/S0140-6736(12)60110-X. 22421340 10.1016/S0140-6736(12)60110-X PMC3316968

