# Supplementary figures and images for: CD300C reduces lung adenocarcinoma susceptibility through regulation of CD62L⁻ monocytes: a Mendelian randomization study
Source: Discov Oncol. 2026 Jan 24;17:326. doi: 10.1007/s12672-026-04481-8 (PMC12913833; doi:10.1007/s12672-026-04481-8)

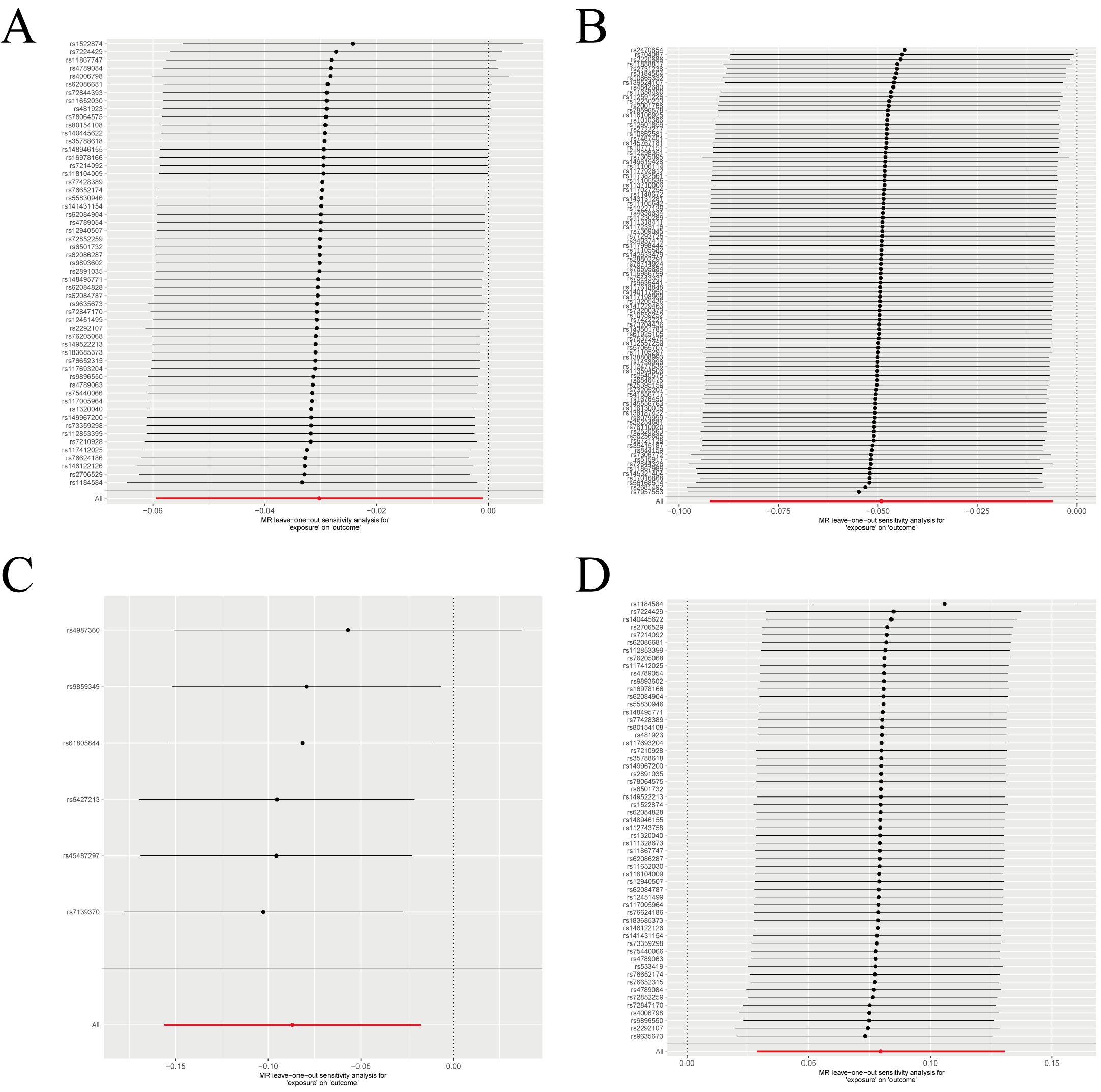

Supplement: Supplementary file 1 — Supplementary Material 1. Figure S1. Leave-one-out MR sensitivity analyses for the associations among CD300C, CD62L⁻ Monocytes, and LUAD. (A) CD300C expression (eQTL-based instruments) → LUAD. (B) CD300C protein levels (pQTL-based instruments) → LUAD. (C) CD300C expression → CD62L⁻ Monocytes. (D) CD62L⁻ Monocytes → LUAD.Each point represents the IVW estimate after exclusion of one SNP, with the dashed line indicating the overall IVW estimate using all SNPs. Across all analyses, exclusion of individual SNPs did not materially alter the effect estimates, suggesting that no single SNP disproportionately drove the associations. [file 12672_2026_4481_MOESM1_ESM.tif]

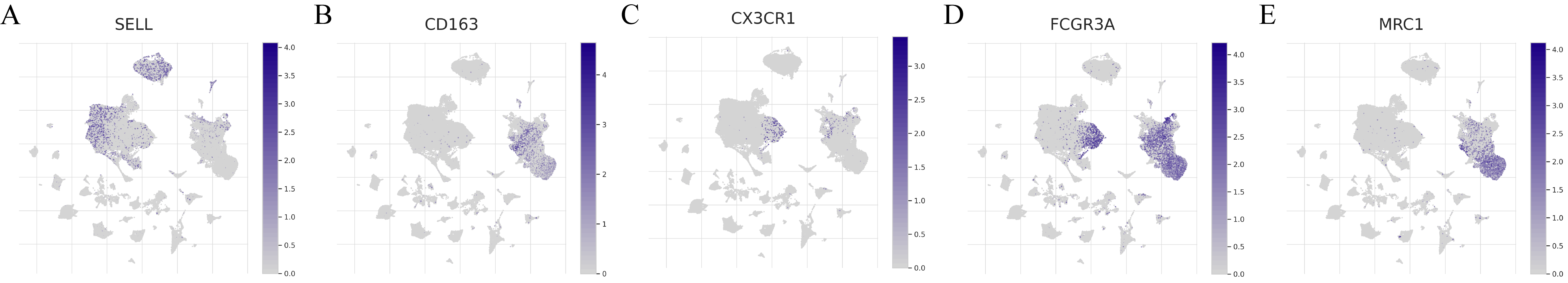

Supplement: Supplementary file 2 — Supplementary Material 2. Figure S2. UMAP visualization of SELL, CX3CR1, FCGR3A, MRC1, and CD163 expression in LUAD single-cell RNA-seq data. [file 12672_2026_4481_MOESM2_ESM.tif]
